# Community service speech language therapists practising in adult dysphagia: Is the healthcare system failing them?

**DOI:** 10.4102/sajcd.v66i1.615

**Published:** 2019-07-18

**Authors:** Kim A. Coutts

**Affiliations:** 1 Department of Speech Pathology, University of the Witwatersrand, Johannesburg, South Africa

**Keywords:** Speech pathology, community service, decision-making, dysphagia, mentoring

## Abstract

Performing community service is a necessity prior to practising speech language therapy in South Africa. This system allows for improved access to these specialised services in the more rural areas. The current challenges of practising in complex settings with little access to mentorship can result in these community service therapists feeling underprepared to assess and manage patients presenting with adult dysphagia (swallowing disorders). This paper explores this topic through the theoretical lens of decision-making, from both clinical and academic perspectives. It aims to pose potential solutions on how to better transform the current practices to be contextually responsive to these challenges.

## Introduction

South Africa’s healthcare system is under considerable stress, as 15% of the healthcare workers need to service 68% of the population within a resource-limited public healthcare setting (Coovardia, Jewkes, Barron, Sanders, & McIntyre, 2009). The role that community service speech language therapists (SLTs) play to bridge this gap for the benefit of the population they serve is of significance; however, I want to pose the question, is the healthcare system failing them? Through the lens of decision-making, this article will highlight the various challenges but also pose potential solutions that can provide development for junior SLTs, with a focus on their practices around adult dysphagia (swallowing disorders).

## Contextual complexity

The South African public healthcare setting is complex. Healthcare institutions range from larger academic or tertiary hospitals with a focus on providing specialised services in the more urban areas and a progressively filter down to primary health care (PHC) clinics that have a focus on more outreach, prevention and basic healthcare services. These institutions are required to function on less than optimal funding to service a large majority of the population, as seen with the lack of funding for permanent posts as reported by Wranz (2011). The current SLT to patient ratio in South Africa can be calculated as 1:18 000 (Health Professions Council of South Africa [HPCSA], 2018). This is a challenging setting for even experienced SLTs.

Compulsory community service for SLTs in South Africa was initiated in 2003, the intention being to provide better access to specialised rehabilitation services for underserved areas of the population (Reid, 2001). It is generally acknowledged that community service is useful for newly qualified therapists, including SLTs for a number of reasons, including that it provides valuable work experience and a unique opportunity to learn and improve their skills and gain knowledge (Wranz, 2011). It also enables them to make a difference in the communities that they serve (Van Stormbroek, 2014).

The challenge is that community service therapists are often placed in rural areas where they are faced with these complex contextual challenges, as well as transitioning into their new role as independent practitioners (Van Stormbroek, 2014). The HPCSA has guidelines for universities that require new SLTs to be able to practise independently (HPCSA, 2012), yet are not able to register as independent practitioners until the completion of their compulsory community service year.

When referring again to adult dysphagia, lack of appropriate clinical expertise can have a serious impact not only for the clinical development of the SLT but for the patient as well, who may receive less than optimal treatment (Singh et al., 2015). Pillay and Andrews (2017) noted that the contextual challenges of limited resources, both human and physical, were a challenge and resulted in long waiting times for the patient and inadequate clinical management of dysphagia cases. This is a daunting task for a junior SLT who is required to practise independently, considering the medical consequences of dysphagia.

Also, South African SLTs students often have limited exposure to dysphagia cases and little experience with objective measures such as videofluroscopy and fibreoptic endoscopic evaluation of swallowing (VFSS and FEES) due to the restricted access to these services within the various training sites, that is, public-sector hospitals and clinics. This can result in community service SLTs feeling somewhat underprepared to practise independently in this area immediately after graduation (Singh, et al. 2015; Yiannopoulos, 2016). When focusing on dysphagia practices, Wranz (2011) explored the readiness, reality and readjustment of community service therapists in South Africa. She noted that while community service SLTs were perceived to have adequate knowledge in the field, they felt inadequate in terms of their skills. Singh et al. (2015) stated that less than half of the sample of community service SLTs felt competent to practise in dysphagia as they lacked confidence, which resulted in feelings of underpreparedness. Based on the information above, to assist community service SLTs they need to develop praxis and build their clinical confidence. The need for mentorship becomes apparent.

## Decision-making frameworks and training in adult dysphagia

Decision-making is a complex process, especially in healthcare. Simplistically, at an undergraduate level, students gain different types of knowledge: theoretical (Patel & Kaufman, 2002) and methodological (Wainwright & McGinnis, 2009). Theoretically, there are limited frameworks that are available to assist in the decision-making processes. There are static one-dimensional models such as those by Yoder and Kent (1980) and Singh (2016). These models are based solely on the clinical signs and symptoms of the patient and lack the impact of a complex contextual setting, as well as the influence of the SLT themselves. The dual processing model suggested by Humbert (2015) is more complex as it involves the metacognitive processes of the SLT but then also lacks the influence of the contextual factors. These models are summarised in Table 1.

**TABLE 1:** Description of the decision-making models and the challenges.

Decision-making model	Yoder and Kent (1988)	Humbert (2016) Dual Processing Theory	Singh (2016)
Description	Algorithm	System One: Based on experience and formal training	Based on a series of options centred on the patient’s signs and symptoms as well
	SLTs can choose management options based on signs and symptoms	System Two: Is analytical and strategic, and involves analysis of all available evidence	-
Challenges	Linear and lacks clinical complexity	Fails to include what information SLTs use from the assessment measure itself and the context in decision-making	Not versatile nor appropriate for the complexity of the settings in which SLTs find themselves.

Note: Although helpful, these models do require revision for use as a guide to SLTs in a setting such as South Africa.

SLT, speech language therapist.

Methodologically, SLTs gain clinical experience and start to integrate the theory with the practical component, called praxis (Whitehead, 2002), which starts at university level but should progress throughout their career. Students in nursing (Banning, 2007), occupational (Reich & Eastwood, 1998) and physiotherapy (Smith & Higgs, 2008) learn clinical decision-making skills through a combination of praxis that is honed through contextual experience and mentorship. These findings corroborate the conclusions from my PhD, which explored the decision-making processes of SLTs assessing dysphagia at the bedside (Coutts, 2017). This study highlighted that the process of praxis is a highly individual one and stems various micro- (intrapersonal) and macro- (external) factors, including their previous clinical experience and exposure during student training. Therefore, limited exposure to dysphagia cases and experience in the more specialised instrumental assessments are critical, often due to lack of availability at various training sites. The importance of having access to appropriate mentorship was also found to be key in this developmental process. The concept of mentorship will be explored in detail in the article.

Another factor that can further impact the decision-making of community service SLTs is the inconsistency of teaching around dysphagia practices in South African universities. Andrews and Pillay (2017) identified the absence of standardised adult dysphagia teaching methods across institutions, which was noted in the different bedside assessment protocols used in clinical practices. These findings were supported by Yiannopoulos (2016), who suggested that South African universities need to standardise their dysphagia curricula to address the new graduates’ feelings of underpreparedness. It is hard to establish sound clinical reasoning and decision-making skills amongst these different practice patterns without appropriate mentorship and senior clinical experience from which to gain knowledge and skills.

In summary, the lack of decision-making models and inconsistency in teachings, coupled with the complexity of practising in the South African healthcare context, highlight the needs for these challenges to be addressed.

## Need for mentorship

There are multiple definitions of mentorship, which describes various roles of what a mentor should encompass. For the purposes of this article, the definition of mentorship in terms of practice, education and research to ensure growth and future of the profession will be the main focus (Gandy, 1993). In South Africa, Schwertdle, Morphet and Hall (2017) mention that due to the complexities of the healthcare system, continuing development is often under-prioritised. If training does occur, the focus is often on senior staff members. This training also takes place in urban areas and is often too expensive to attend, thus making it impractical for many community service SLTs. This needs to change.

The transition from student to a practising clinician is a complex one (Van Stormbroek, 2014). The process starts with enthusiasm and excitement but quickly dissipates when various challenges of the context are realised. The study on South African community service occupational therapists learning a specialised skill (Van Stormbroek, 2014) revealed interesting results that may also be generalised to SLTs. It showed that the reality of practice was responded to with a process of adaptation, where the therapists learn to respond to the realities of the context (Van Stormbroek, 2014). In South Africa, community service therapists start to shift their perception from their ability and skill set as theoretical, to needing to meet the needs of patients more clinically (i.e. praxis). Some therapists also struggle with developing their clinical reasoning skills (i.e. decision-making abilities) and adopting a client-centered approach due to the various contextual challenges (Van Stormbroek, 2014). This lack of confidence that the therapists experienced was in direct correlation to their lack of support and led to other feelings of anxiety, frustration and inadequacy (Van Stormbroek, 2014). A key finding from Van Stormbroek (2014) was that the biggest advantage in assisting with the transition to independent clinician was that the students received the support that they needed. All participants in her study felt that they needed access to senior staff for mentorship, which may be expected to be the case with SLTs.

Wranz (2011) showed that although community service SLTs felt generally underprepared, their overall clinical skills did improve over the course of their community service year, which is encouraging. However, dysphagia was one of the main areas in which all SLTs still do not feel confident to practise independently due to the medical risks, which include aspiration, dehydration, malnutrition and possible death (Bax, MacFarlane, & Green, 2014). The SLT participants stated that they felt more alone during their community service year, as there was either no or very limited access to the necessary supervision or support, even from peers. Wranz (2011) also noted that the true development of knowledge and skills is greatly improved when newly qualified SLTs receive adequate mentoring under appropriate supervision, and that the absence of mentors in the South African context was a grave concern. This was reported in the findings from the participants in her study.

In the studies mentioned above, there appeared to be little access to appropriate supervision, either because there were no other SLTs available, or the colleagues were also inexperienced. This stems from the broader challenge of the lack of funds for posts in the public healthcare sector. Although being in a working environment will inevitably result in skill development, the SLTs are unlikely to learn as much as if they were under mentorship. This is particularly pertinent for dysphagia, where serious adverse consequences can arise due to a lack of knowledge and skills.

There are significant benefits to mentorship as noted from a variety of healthcare professions. These benefits include: (1) Under appropriate mentorship, they had the freedom and support to make mistakes and to then learn from them (Van Stormbroek, 2014); (2) Naidoo (2006) stated that in physiotherapy, it creates dialogue about practice, promotes development, stimulates reflection, yielded productivity and refined leadership skills; (3) Henry-Noel, Bishop, Gwede, Petkova and Szumacher (2018) noted that mentorship is an essential process to academic medicine and promotes professional satisfaction; (4) In speech therapy, Pamploma, Ysunza, Sarre, Morales and Sterling (2015) stated that mentorship aided in self-confidence and overall performance; (5) Wranz (2011) said it well when the first few years of a career are vital for developing good habits, favourable attitudes and proper work ethics, which can only come from adequate mentorship.

All of these skills are important contributors to positively impacting on the overall decision-making processes of SLTs in adult dysphagia in the complex public healthcare context.

## Potential solutions

There has been a recent cry for the transformation of practices from the South African student bodies, and these potential solutions can assist in starting this process.

In New Zealand, the United States and Ireland, new graduates are supervised for a certain period of time (IASLT, 2015) prior to their registration as independent practitioners. This is also the case as seen with the medical doctors who completed 2 years of internship prior to entering their community service year. Potentially, an internship model could be proposed for SLTs prior to expecting community service SLTs to practise independently in such challenging circumstances.

Five studies (Coutts, 2017; Pillay & Andrews, 2017; Singh et al., 2015; Van Stormbroek, 2014; Wranz, 2011) suggested that the development of knowledge and skills in adult dysphagia can continue with the attendance of continuing professional development courses, even in the absence of supervision. Attending courses allow junior SLTs to engage with the latest clinical information and to learn from senior colleagues, but it is not a long-term solution. These Continuing Professional Development (CPD) courses also need to be accessible and affordable for community service SLTs from all settings.

There needs to be a creative collaboration between the Department of Health and the Department of Higher Education that focuses on junior colleagues being able to access mentors in the profession from both clinical and research perspectives. University staff members can create platforms using a blended learning approach (i.e. Skype) to assist SLTs in continuing professional growth and support from all contexts. This is important to combat the limited number of available staff in the Department of Health who cannot take on the active role of mentorship, considering their case load and often inaccessibility.

Future research is required in establishing how a mentorship programme from both within and external to the Department of Health can be established and potentially benefit the skills and development of junior SLTs in adult dysphagia specifically.

## Conclusion

Both locally and abroad, having novice SLTs practising in under-resourced settings is inevitable, and it is of vital importance that they are adequately prepared for this year (Van Stormbroek, 2014). In the context of the lack of adequate mentorship and complex contextual challenges, making decisions in adult dysphagia independently can be daunting for many SLTs, which can have serious clinical consequences. There are potential solutions in which higher education institutions need to ensure that they are able to provide the support that bridges this gap by providing mentorship and access to other learning opportunities. The solution will require creative collaboration by the Department of Health and Department of Higher Education. These solutions can further assist in starting to answer the call for transformation of our practices within the profession.
